# Structures and Divergent Mechanisms in Capsid Maturation and Stabilization Following Genome Packaging of Human Cytomegalovirus and Herpesviruses

**DOI:** 10.3390/life11020150

**Published:** 2021-02-16

**Authors:** Clotilde Muller, Sophie Alain, Thomas F. Baumert, Gaëtan Ligat, Sébastien Hantz

**Affiliations:** 1 INSERM, CHU Limoges, University of Limoges, RESINFIT, U1092, 87000 Limoges, France; clotilde.muller@unilim.fr (C.M.); sophie.alain@unilim.fr (S.A.); 2 CHU Limoges, Laboratoire de Bactériologie-Virologie-Hygiène, National Reference Center for Herpesviruses (NRCHV), 87000 Limoges, France; 3 Institut de Recherche sur les Maladies Virales et Hépatiques, Université de Strasbourg, 67000 Strasbourg, France; Thomas.Baumert@unistra.fr; 4 Institut Hospitalo-Universitaire, Pôle Hépato-Digestif, Nouvel Hôpital Civil, 67000 Strasbourg, France

**Keywords:** herpesviruses, human cytomegalovirus, capsid maturation, DNA packaging, therapeutic targets

## Abstract

Herpesviruses are the causative agents of several diseases. Infections are generally mild or asymptomatic in immunocompetent individuals. In contrast, herpesvirus infections continue to contribute to significant morbidity and mortality in immunocompromised patients. Few drugs are available for the treatment of human herpesvirus infections, mainly targeting the viral DNA polymerase. Moreover, no successful therapeutic options are available for the Epstein–Barr virus or human herpesvirus 8. Most licensed drugs share the same mechanism of action of targeting the viral polymerase and thus blocking DNA polymerization. Resistances to antiviral drugs have been observed for human cytomegalovirus, herpes simplex virus and varicella-zoster virus. A new terminase inhibitor, letermovir, recently proved effective against human cytomegalovirus. However, the letermovir has no significant activity against other herpesviruses. New antivirals targeting other replication steps, such as capsid maturation or DNA packaging, and inducing fewer adverse effects are therefore needed. Targeting capsid assembly or DNA packaging provides additional options for the development of new drugs. In this review, we summarize recent findings on capsid assembly and DNA packaging. We also described what is known about the structure and function of capsid and terminase proteins to identify novels targets for the development of new therapeutic options.

## 1. Introduction

Human herpesviruses are large and structurally complex viruses divided into three subfamilies: *Alphaherpesvirinae* such as herpes simplex virus types 1 and 2 (HSV-1 and -2) and varicella-zoster virus (VZV); *Betaherpesvirinae* such as human cytomegalovirus (HCMV) and human herpesvirus types 6A, B and 7 (HHV-6A, -6B and -7); and *Gammaherpesvirinae* such as EBV (Epstein–Barr virus) and human herpesvirus 8 (HHV-8). They are responsible for a wide variety of pathologies ranging from simple localized lesions to disseminated damages. The severity of the clinical forms and the extension of the lesions are closely linked to the immunosuppressive state of the patients. Alphaherpesviruses cause a variety of diseases ranging from cutaneo-mucosal vesicles to encephalitis or disseminated infections. Betaherpesvirus primary infections are often asymptomatic in immunocompetent patients, but viral reactivation can lead to life-threatening complications in immunocompromised individuals. For instance, HCMV is responsible for severe diseases in immunocompromised patients. Moreover, HCMV is the most common infectious cause of congenital malformations, with developmental delay, sensorineural hearing loss and fetal death in 10–15% of cases [1]. Gammaherpesvirus primary infections are also often mildly symptomatic, except for infectious mononucleosis, but EBV can induce lymphoproliferative disorders, and HHV-8 Kaposi sarcoma.

The approved treatment for HSV-1 and HSV-2 infections is acyclovir. Ganciclovir, valganciclovir, cidofovir and foscarnet are used for the treatment of HCMV infections. These licensed molecules share the same mechanism of action of targeting the viral DNA polymerase. Limitations of these drugs are their dose-limiting toxicity and resistance emergence, leading to therapeutic challenges [2,3,4]. Letermovir (AIC246) has recently been shown to be effective against HCMV infections. It is indicated for the prophylaxis of HCMV reactivation and HCMV disease in HCMV-positive adults receiving allogeneic hematopoietic stem cell transplantation. However, this new drug has no significant activity against other herpesviruses or nonhuman CMV, and resistance mutations have already emerged [5,6]. Letermovir acts via a novel, not fully understood mechanism. This drug inhibits the cleavage of viral DNA concatemers and the formation of mature HCMV virions by targeting the pUL56 subunit of the viral terminase complex [5,6]. Mutations associated with letermovir resistance map mainly to pUL56. To date, no clinical treatment is available for EBV or HHV-6, -7 and -8.

**Figure 1:**
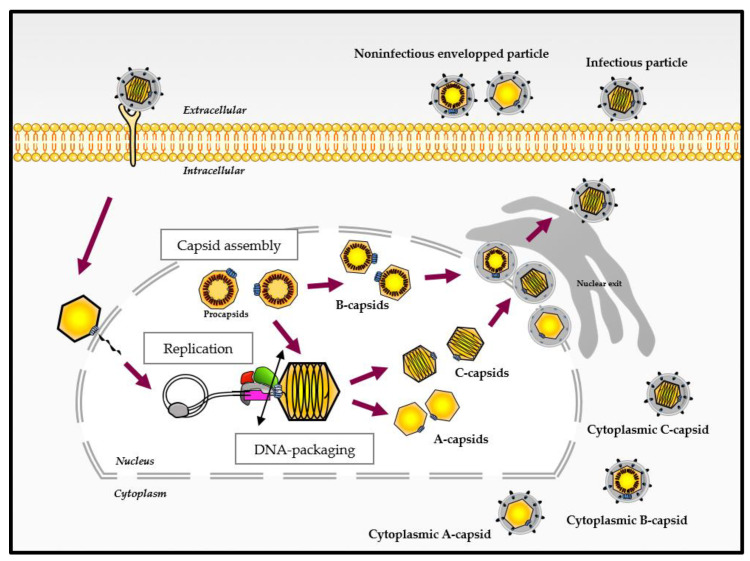
Different virus-like particles during the viral cycle of herpesviruses (adapted from [5,6]). After binding and entry into the host cell, the capsid is transported to the nuclear pore and delivers viral DNA into the nucleus. After genome replication, the DNA packaging step occurs and ends with the cleavage of the concatemers (represented by the double-ended black arrow), releasing viral DNA into the capsid. Different capsid forms are present in the host cell nucleus during infection. These capsid forms, referred to as A-, B- and C-capsids, represent empty capsids, scaffold-containing capsids and viral DNA-containing capsids, respectively. The C-capsids are considered as a precursor of infectious virus.

The infection cycle of HCMV, as for other *Herpesviridae*, comprises various steps in the cell nucleus where genome replication and the assembly of capsids take place. The replication of the 230 kbp DNA genome is thought to occur by a rolling circle process and results in the formation of concatemers that are cleaved into unit-length genomes and packaged into a preformed capsid (Figure 1). The capsid assembly and DNA packaging are crucial steps for herpesvirus multiplication involving various viral proteins. Importantly, these steps are highly specific to the herpesvirus family, have no counterpart in the human organism and thus represent a target of choice for the development of new antivirals.

There remains an unmet need to develop new therapeutic strategies for herpesvirus infections targeting other replication steps than DNA replication and inducing fewer adverse effects. Addressing this issue, in this review, we will discuss recent findings on HCMV and herpesvirus capsid assembly and DNA packaging and further explain the potential of capsid and terminase proteins as new antiviral targets for the development of new effective treatments for HCMV infections.

## 2. Assembly of Herpesvirus Capsids

**Figure 2:**
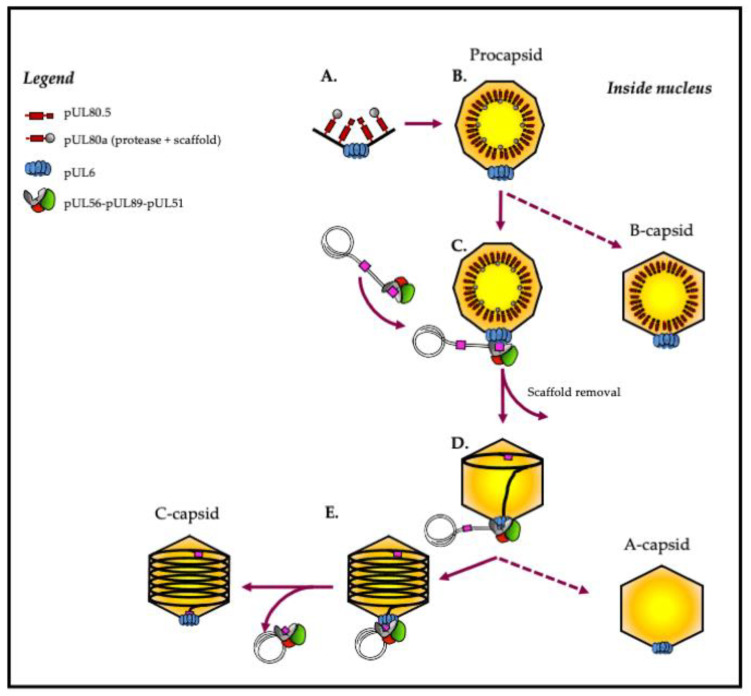
Capsid formation and DNA packaging of herpesviruses: model of HCMV. (**A**,**B**) The intranuclear capsid formation is initiated by the assembly of capsid proteins comprising MCP (black), SCP (black), triplexes (black), portal proteins (blue) and scaffold proteins (red and grey). (**C**) Maturation of the nucleocapsid is achieved by activating the maturational protease, which performs proteolytic digestion of the scaffold. At the same time, the terminase complex recognizes the “pac” motifs (“cis-acting packaging signal”) represented in pink. (**D**) The complex then performs an initial cleavage of viral DNA and exerts its ATPase activity to power the translocation of a unit-length DNA genome into the nucleocapsid. (**E**) Terminase proteins perform a second cleavage and release viral DNA into the capsid. Finally, the complex dissociates from the filled capsid and is ready for the next DNA-packaging step.

The capsid structure of herpesviruses was resolved by cryo-electron microscopy, which confirmed that the HCMV three-dimensional capsid structure is similar to the alpha- and gamma-herpesviruses’ capsid structure in overall organization [7,8]. The HCMV genome is twice the size of the VZV genome and >50% larger than the HSV-1 genome [9]. Despite enclosing a much larger genome, the size of the HCMV capsid is similar to that of HSV-1 and to those of other herpesviruses, which range around a diameter of 100 nm [10]. Infectious herpesvirus virions have an icosahedral capsid with a triangulation number (T) of 16, composed of 150 hexons that constitute the 30 edges and 20 faces of the icosahedron, with 11 pentons occupying 11 of 12 vertices and 320 triplexes that link the pentons and hexons together. Proteins forming an annular structure named the ‘portal’ occupy the twelfth vertex [11]. The procapsid is a complex two-shell structure containing various proteins, including the major capsid protein (MCP) and the smallest capsid protein (SCP) (Table 1). Each protein is specifically designed to associate with the final structure in a precise manner [11] (Figure 2).

### 2.1. The Major Capsid Protein (MCP)

**Figure 3:**
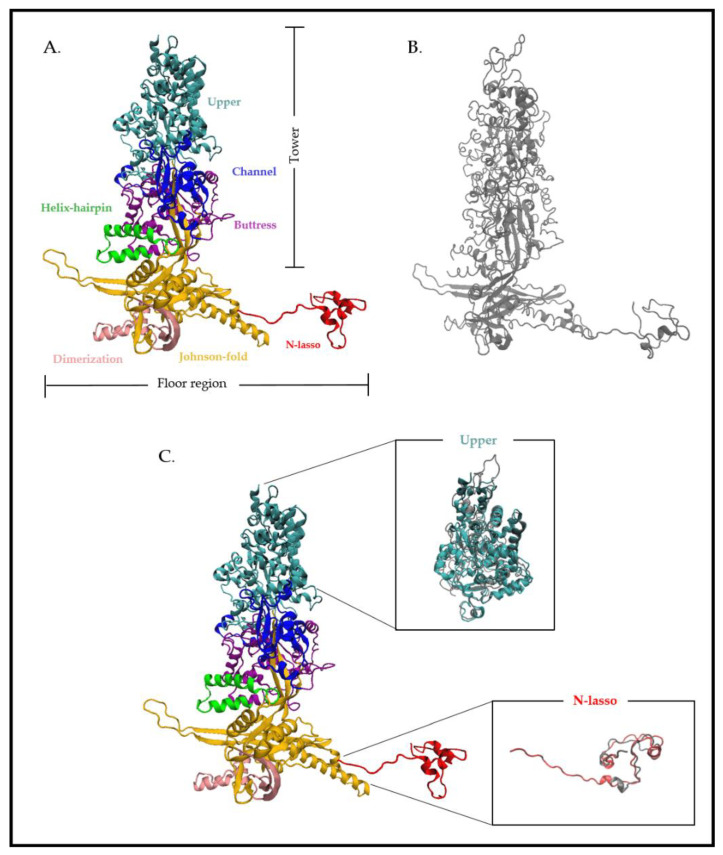
Atomic structure of the HCMV major capsid protein (MCP) pUL86 and the HSV-1 MCP VP5. (**A**) Cryo-electron microscopy structure of the HCMV MCP pUL86 (each domain is highlighted in different colors). (**B**) Cryo-electron microscopy structure of the HSV-1 MCP VP5. (**C**) Partial superposition of the upper and N-lasso domain of pUL86 and VP5. Superposition of the proteins shows a higher atomic divergence for both the upper and N-lasso domains. The analysis is based on the HCMV and HSV-1 capsid structure (PDB: 5VKU and 6CGR) using Visual Molecular Dynamics (VMD 1.9.3).

The most abundant protein components of the outer shell are the MCPs. Capsids comprise 11 pentons and 150 hexons that are divided into five and six MCP copies, respectively. In HCMV, the viral gene *UL86* encodes the major capsid protein pUL86, composed of 1370 amino acids (Figure 3). pUL86 is essential for HCMV capsid formation and the generation of viral progeny. This is one of the most conserved proteins in herpesviruses [12] (Figure 3, Table 1). The MCP domains are organized into two regions formed by distinct domains (Figure 3): a tower region composed of the upper (amino acids (aa) 481 to 1031), channel (389 to 480 and 1322 to 1370), buttress (1107 to 1321) and helix-hairpin (190 to 233) domains, which make up the penton and hexon capsomer protrusions, and a floor region composed of the N-lasso (1 to 59), dimerization (291 to 362) and Johnson-fold (60 to 189, 234 to 290, 363 to 397 and 1032 to 1106) domains [13]. Superposition of the cryo-electron microscopy structure of HCMV MPC (pUL86) and HSV-1 MCP (VP5) has shown strong similarities. Differences observed are more pronounced in the N-lasso and upper domain, in particular at the top of the upper domain where SCP binding occur (Figure 3). This similarity of secondary and tertiary structures was also demonstrated through structural alignments based on the VP5ud model and the HCMV MCP model’s upper domain [9]. For HHV-8, although the structures are similar, a loop in the HSV-1 MCP is replaced by a helix in HHV-8, producing a groove into which an SCP binds [13].

### 2.2. The Smallest Capsid Protein (SCP)

The *UL48.5* gene encodes the smallest HCMV protein. This protein of 75 amino acids, essential for HCMV viral growth, would participate in the cohesion of the capsid by coating the ends of the hexons and pentons [14]. Unlike in HCMV, where SCPs bind hexons and pentons so that exactly one SCP binds each MCP, the HSV-1 SCP binds only hexon MCPs [9,15]. Moreover, the SCP in HSV-1 is nonessential for viral growth in cell culture [16].

### 2.3. External Scaffolding Proteins: Triplex Proteins

The triplexes sit atop the MCP N-lasso triangle and are critical for capsid morphogenesis, most likely linking pentons and hexons together, directing assembly and stabilizing the structure prior to maturation. There are 320 of them in the capsid. Triplexes are heterotrimers consisting of two dimer-forming parts of the minor capsid protein (mCP), coupled with a single copy of the minor capsid binding protein (mCP-bp) [14]. The mCP of HCMV, pUL85, is a protein composed of 306 amino acids that forms dimers [9] (Table 1). The HCMV mCP-bp is encoded by the *UL46* gene (Table 1).

### 2.4. Maturational Protease and Internal Scaffolding Protein from the Fusion Protein

In addition to the structural proteins, capsid assembly involves the formation of scaffold proteins for the generation of the capsid subunit and the procapsid structures. The scaffolding proteins of HCMV have been identified as gene products of the *UL80a* and *UL80.5* open reading frames (ORFs) [17]. *UL80a* encodes the assembly protein precursor, which is proteolytically processed into protease and scaffold domains. The scaffold domain corresponds to the C-terminus (C-term; pUL80, aa 336 to 708) and the protease (also called assemblin, pUL80a) to the N-terminus (N-term; pUL80, aa 1 to 256). The protease is subsequently autocatalytically cleaved into two more peptides [17]. Three homologs are reported in HSV-1, VP21 (pUL26), VP24 (pUL26) and VP22a (pUL26.5) [18] (Table 1). The assembly protein, encoded by *UL80.5*, directly interacts with the MCP (pUL86) [19]. Importantly, the MCP–assembly protein interaction is essential for the nuclear translocation of the MCP [20]. A conserved domain in the N-term of pUL80.5 promotes self-interaction and has been suggested to lead to the generation of multimers [20]. The self-interactions together with the MCP is thought to catalyze the formation of intranuclear hexons and pentons [21]. The HSV-1 homolog *UL26.5* encodes preVP22A, which interacts with the HSV-1 MCP (VP5) through its C-term 25 amino acids that remain inside A-, B- and C-capsids [22]. Specifically, 12 hydrophobic amino acids at this C-term end are critical for the interaction, suggesting that the preVP22A–VP5 interaction is a hydrophobic interaction [22].

Although the scaffold plays a central role in capsid assembly, to date, no scaffolding protein has been found within the mature capsid or the virion (Figure 2). *UL80a* encodes the assembly protein precursor composed of the protease (pUL80a) and the assembly protein (pUL80.5). Like HCMV, the HSV-1 pUL26 is initially extended at its N-term by the protease, and a linker and its C-term part is identical to the major form, pUL26.5 [23]. This fusion provides a convenient mechanism for incorporating the protease into the assembling procapsid. The final step of the capsid maturation in HCMV and HSV-1 involvs a proteolytic cleavage for the MCP binding domain of pUL80.5 and preVP22a, respectively [24,25]. Bioinformatics analysis has identified a protein family predicted to share the canonical herpesvirus protease fold, having conserved Ser and His residues in their active sites [23].

### 2.5. The Portal Protein

The capsid is also composed of a portal that occupies one of the 12 vertices of the capsid and plays a critical role for herpesvirus replication. This arrangement of 12 monomers forms a ring, allowing herpesvirus DNA to be incorporated into the preformed capsid. Portal proteins act as the docking site for the terminase–DNA complex. pUL104 is the HCMV dodecameric portal protein, with 12-fold symmetry. pUL104 directly interacts with the large terminase subunit pUL56. This interaction was shown to be essential for DNA insertion into the capsid [26]. pUL6, the HSV-1 portal protein, is one of the most studied portal proteins in herpesviruses, and its structure was recently resolved using cryo-electron microscopy [27]. Structurally, a portal monomer consists of five domains; the wing, stem, clip, β-hairpin and wall [27]. The twelve monomers are arranged such that their loop-rich wing domains form the outer periphery of the complex. The remaining stem, clip, β-hairpin and wall domains line the interior of the portal’s DNA translocation channel. The formation of a stable ring requires a putative leucine zipper, as well as disulfide bonding between subunits [28,29]. It has been shown that the portal protein pUL6 interacts with the scaffolding protein VP22a. This interaction requires a region corresponding to amino acids 143 to 151 of VP22a and is essential for viral replication, DNA cleavage/packaging and incorporation of the portal into capsids [11].

## 3. Viral DNA Cleavage/Packaging

The replication of HCMV DNA initiates from the HCMV DNA replication origin and forms concatemers by rolling circle-type replication. The DNA packaging and maturation are two steps that take place simultaneously in the nucleus. The DNA-packaging process happens as follows [30]: (1) After translocating into the nucleus, (2) the terminase complex recognizes the cis-regulatory “*pac*” (“cis-acting packaging signal”) motifs located in the “*a*” part of the HCMV genome and recruits a procapsid. (3) The complex then performs an initial cleavage of the viral DNA (4) and exerts its ATPase activity to power the translocation of a unit-length DNA genome into the procapsid. (5) HCMV terminase proteins then perform a second cleavage, releasing viral DNA into the capsid via an interaction with pUL104 protein. (6) Finally, the complex is ready for the next DNA-packaging step (Figure 1 and Figure 2). In general, terminase complexes are hetero-oligomers composed of three core proteins (pUL56, pUL89 and pUL51 for HCMV and pUL28, pUL15 and pUL33 for HSV-1), each carrying a different function required for the packaging process [30,31,32]. At least three other proteins (pUL52, pUL77 and pUL93 for HCMV and pUL32, pUL25 and pUL17 for HSV-1) seem to be part of the terminase complex and/or participate in the DNA cleavage/packaging process [32,33,34,35,36].

### 3.1. The Terminase Large Subunit

**Figure 4:**
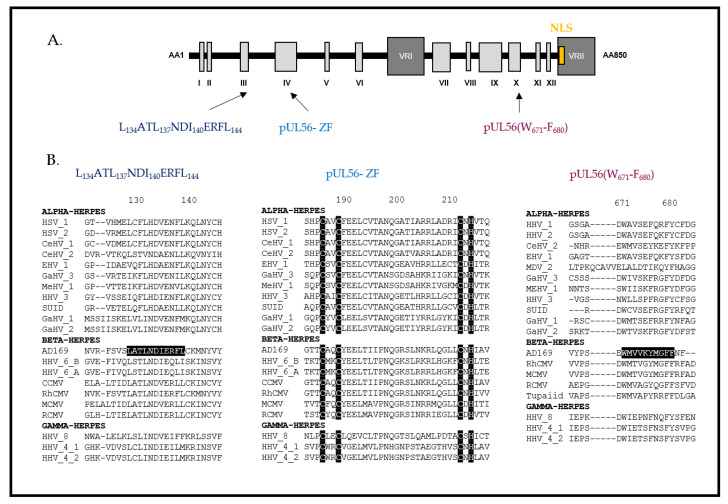
Structure of the HCMV terminase subunit pUL56 (adapted from [37,46]). (**A**) pUL56 is composed of 12 conserved (I–XII) and 2 variable regions and comprises 3 essential domains annotated LATLNDIERFL, pUL56-ZF (zinc finger) and pUL56 (W_671_–F_680_). (**B**) Sequence alignments of conserved regions from various herpesviruses belonging to the α, β and γ subfamilies of herpesviruses. The nuclear localization signal (NLS) of pUL56 is shown by a yellow box at the amino acid position 816 to 827. Sequence numbering is consistent with that of the HCMV reference strain AD169 residues. Key residues involved in the formation of the three patterns are shown as white letters on a black background.

**Figure 5:**
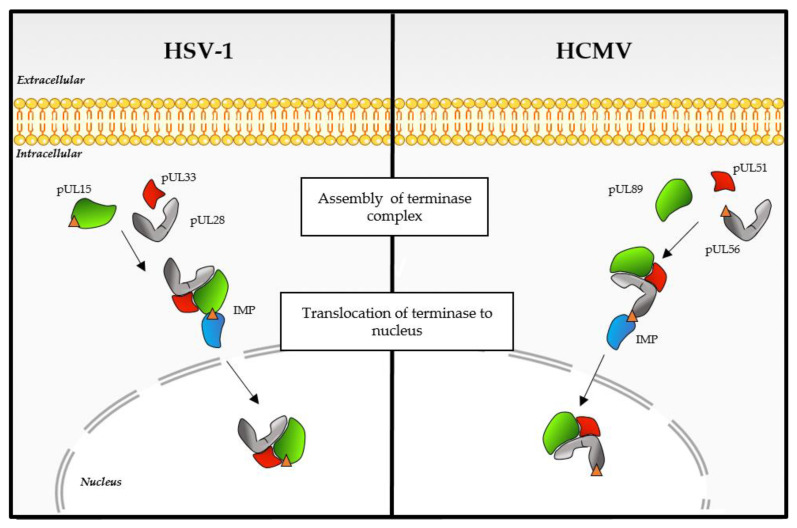
Nuclear import of the terminase complex: model for HSV-1 and HCMV. The terminase complex is represented as follows: pUL56 (HCMV) and pUL28 (HSV-1) are in grey, pUL89 (HMCV) and pUL15 (HSV-1) are in green and pUL51 (HCMV) and pUL33 (HSV-1) are in red. The importin (IMP) is represented in blue, and the nuclear localization signal (NLS) is shown as an orange triangle. Once the complex is associated, its translocation in the nucleus is mediated via the importin-dependent pathway.

The large subunit pUL56 is a protein composed of 850 amino acids and 12 conserved regions [37] and is encoded by the ORF *UL56* located on the unique long region of the HCMV genome (Figure 4). pUL56 is a homolog of the HSV-1 protein pUL28, which has an essential role in HSV-1 genome packaging [38,39]. The predicted molecular weight of pUL56, based on amino acid composition, is 96 kDa. However, some studies, including our personal experiments, have shown that it migrates with a higher apparent molecular weight on Western blots (130 kDa). pUL56 is involved in the ATPase activity of the complex and it specifically recognizes and interacts with the “*pac*” motifs [30]. The presence of a nuclear localization signal (NLS) at the amino acid position 816–827 was validated in pUL56 by Giesen et al. [40]. Sequential substitutions by alanine scanning indicated the importance of the residues R and K at positions 822 and 823. In addition to allowing the translocation of pUL56 in the nucleus via the importin-dependent pathway, this NLS also allows the transport of the whole complex (pUL89–pUL56–pUL51) [40] (Figure 5). The specific recognition of these motifs is necessary for the cleavage of a genomic unit. Studies also demonstrated that pUL28 interacted with the “*pac*” motif [41]. The suspected ATP binding site is located in the C-term half of pUL56 and involves the amino acids _709_YNETFGKQ_716_ (in HCMV pUL56) [42,43]. A specific interaction between the C-terminal part of pUL56 and pUL89 was also detected by coimmunoprecipitation [44]. Importantly, we have shown that a short sequence of ten residues located in the C-terminal region of pUL56 (_671_WMVVKYMGFF_680_ in HCMV pUL56) is essential for this interaction [45] (Figure 4). Recently, we suggested that pUL56 belongs to the LAGLIDADG homing endonucleases family. This recent finding was supported by the identification of a LATLNDIERFL and a zinc finger pattern in the N-term of pUL56 essential for viral replication [46] (Figure 4). We thus proposed that two subunits of pUL56 use the LATLNDIERFL pattern as an interface domain where the hydrophobic amino acids L134, L137, I140 and L144 dimerize. The acidic residue E141 may contribute to the nuclease sites. Residues C191, C194, C217 and H219 in HCMV pUL56 or C197, C200, C223 and H225 in HSV-1 pUL28 are conserved and most likely correspond to the zinc coordinating amino acids within the zinc-finger pattern (Figure 4). Interestingly, the LATLNDIERFL and zinc-finger patterns are located near the pUL56 region encompassing mutations that facilitate letermovir resistance. Thus, these motifs could be involved in the mechanism of action of letermovir [46]. However, for most of the identified patterns, functional studies are needed to investigate their function in the viral life cycle.

### 3.2. The Terminase Small Subunit

**Figure 6:**
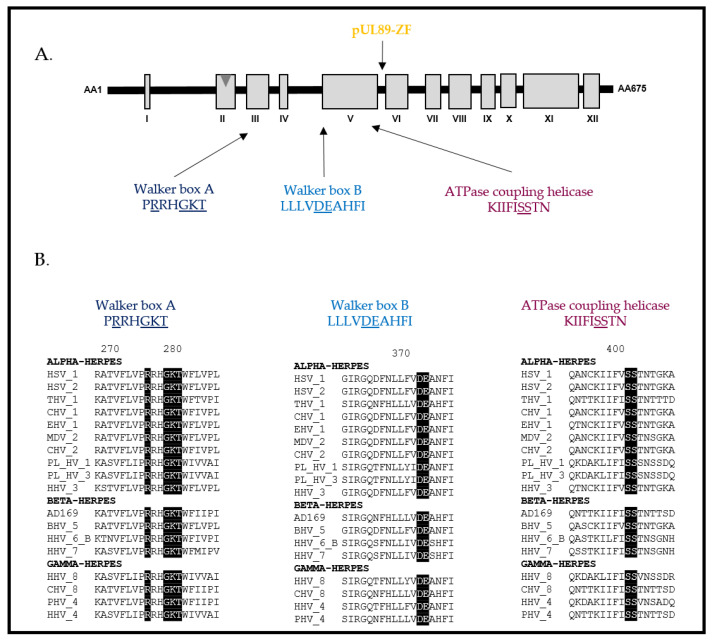
Structure of the HCMV terminase subunit pUL89 (adapted from [37,46]). (**A**) pUL89 is composed of 12 conserved regions (I–XII) and comprises putative functional domains such as the pUL89 zinc-finger domain, annotated pUL89-ZF, and the adenine binding site (Walker A box, Walker B box and ATPase coupling helicase). Underlined amino acids are residues involved in the activities of each domain. (**B**) Sequence alignments of conserved regions from various herpesviruses belonging to the α, β and γ subfamilies of herpesviruses. Sequence numbering is consistent with that of the HCMV reference strain AD169 residues. Key residues involved in the formation of the three patterns are shown as white letters on a black background.

The small subunit pUL89 is a protein of 674 amino acids encoded by the ORF *UL89* located on the unique long region of the HCMV genome (Figure 6). pUL89 is a homolog of the HSV-1 protein pUL15. This protein of approximately 75 kDa possesses a putative nuclease domain and a putative DNA binding domain, implying that the aspartate 463 is involved in nuclease activity and the arginine 544 in DNA binding [31]. Different conserved patterns were highlighted in silico, comprising an adenine binding site (_156_EPFQ_159_ in HCMV pUL89), a Walker A motif (_213_PRRHGKT_219_ in HCMV pUL89), a Walker B motif (_305_LLLLVDEAHFI_314_ in HCMV pUL89) and pattern III (_337_SST_339_ in HCMV pUL89) [47] (Figure 6). To date, pUL89 has only been shown to have in vitro nuclease activity and a 30% increase in the ATPase activity of pUL56 when they are interacting [42,48,49]. Whereas the HSV-1 homolog pUL15 showed an NLS that gives the ability to enter the cell nucleus independently [50], the nuclear localization of HCMV pUL89 and pUL51 required three subunits [51] (Figure 5). For pUL56, as for most of the identified patterns, functional studies are needed to investigate its function in the viral cycle.

### 3.3. Additional Proteins Contribute to the HCMV DNA Cleavage/Packaging Process

Unlike bacteriophages, which have a large subunit (Lter) and a small subunit (Ster), herpesviruses have more proteins involved in the terminase complex. At least three additional HCMV proteins, namely pUL52, pUL77 and pUL93, contribute to the DNA cleavage/packaging process [32,33,34,35,36]. These proteins are homologous to the HSV-1 proteins pUL33, pUL32, pUL25 and pUL17, respectively. The *UL51* gene encodes a protein of 157 amino acids that is crucial for DNA cleavage/packaging. pUL51 interacts with the HCMV terminase subunits pUL56 and pUL89 and mediates their correct subnuclear localization [32]. Borst et al. reported that the C-terminal part of pUL51 carries an essential function for the growth of the virus. They have shown that the C-terminal part of pUL51 is sufficient to transport the assembly of the terminase complex (pUL56–pUL89–pUL51) and promote its correct nuclear localization [52]. pUL52 does not seem to be involved in the nuclear localization of the terminase complex. Deletion of this cysteine-rich protein leads to uncleaved HCMV DNA, suggesting that pUL52 is crucial for the HCMV DNA cleavage/packaging process [33]. Although pUL52 is not detected in viral replication compartments, a time-course experiment with pUL32, the HSV-1 homolog of HCMV pUL52, revealed a dynamic localization pattern. Early in the course of infection, pUL32 was found predominantly in replication compartments, while later, its localization became diffuse within the nucleus and the cytoplasm [53]. The precise function of pUL52 remains unknown. However, Albright et al. suggested that the HSV-1 homolog may act to modulate disulfide bond formation during procapsid assembly and maturation. The essential HCMV protein encoded by *UL77* has been shown to interact with many proteins involved in capsid formation, such as the MCP, tegument protein pUL71, terminase complex, portal protein and pUL93, as well as the dsDNA. Although its role has not yet been elucidated, this protein participates in the stability of the entire capsid and plays a key role during viral capsid formation [54,55]. In addition, its HSV-1 pUL25 homolog also seems to be necessary to maintain the stability of the capsid and multiple interactions were also highlighted [56]. pUL93 interacts with pUL77 and components of the nuclear egress complex (pUL50–pUL53–pUL97) and seems to play a key role during viral capsid formation [35,36,57]. Recently, it was shown that pUL17 (pUL93 in HCMV) anchors the terminase complex to the capsid and is involved in determining the site of the first cleavage reaction. In addition, pUL17 interacts with the asymmetric dimer of pUL25 (pUL77 in HCMV) [58].

## 4. Different Capsid Forms Are Present in the Host Cell: A-, B- and C-Capsids

As mentioned above, the procapsid contains a protease that is crucial for capsid maturation. For all herpesviruses, the protease is activated at the time of DNA packaging, and its cleavage causes a significant rearrangement of the procapsid. The outer shell hence changes from a roughly spherical to an icosahedral shape [11]. The procapsid, the first closed spherical structure formed during the assembly process, is a precursor form of three other capsid types [59]. During infection, three types of capsids are detected within the host cell (Figure 1). These capsids forms, namely A, B and C, represent empty capsids, scaffold-containing capsids and viral DNA-containing capsids, respectively. The morphology of the capsid remains similar regardless of its type, but the protein composition differs on the inner and outer shells of the capsid [9,27,59,60].

C-capsids are identical or at least similar to those found in mature virions. They represent the procapsids that have correctly packaged the viral genome during angularization of the capsid [59]. A-capsids are essentially hollow and do not contain DNA or scaffolding proteins. This type of capsids probably arises after partial or complete DNA packaging [59]. B-capsids contain internal scaffold proteins (i.e., VP22a and VP21 for HSV-1)**,** suggesting that the capsid is either preparing for the packaging step or that the scaffold was not removed in time to allow for DNA packaging [61].

Both A- and B-type HCMV capsids are considered as dead-end products. While all three capsid forms are present in the nucleus and in the cytoplasmic compartments, C-capsids predominate in the cytoplasm. When released from the cell, A- and B-capsids appear to constitute a population called noninfectious enveloped particles [61].

## 5. Inhibitors of Terminase and Capsid Proteins for the Treatment of Herpesvirus Infections

Due to their crucial role in the formation of new infectious virions and the lack of counterparts in mammalian cells, the capsid assembly and DNA-packaging steps can be explored for the development of new therapeutic approaches. Targeting viral proteins involved in these both steps has been shown to be an interesting approach for the development of new therapeutic strategies.

Drugs based on the benzimidazole chemical backbone are nucleoside analogs active against HCMV that were originally synthesized by Townsend et al. [62]. Addition of bromine to the 2-position of the core 5,6-dichloro-1-β-D-ribofuranosyl benzimidazole (DCRB) structure yields analogs such as BDCRB (2-bromo-5,6-dichloro-1-β-D-ribofuranosyl benzimidazole) and TCRB (the 2-chloro analog of BDCRB) [63]. The primary mechanism of BDCRB function involves inhibition of the formation of HCMV unit-length genomes. Genetic mapping experiments showed that inhibition of viral DNA maturation is mediated by interactions involving the products of the HCMV *UL89* and *UL56* ORFs [64]. The GlaxoSmithKline compound GW-275175X also targets this step and has a longer plasma half-life [63]. BAY 38-4766, also called tomeglovir, is an inhibitor of HCMV replication. This nonnucleoside inhibitor appears to target the proteins pUL89 and pUL104. Resistance mutations in these two proteins were observed after analysis of resistant strains [65]. Another molecule, WAY-150138, is an N-(4-(3-[5-chloro-2,4-dimethoxyphenyl]-thioureido)-phenyl) acetamide-targeting pUL6 of herpes simplex virus. This molecule inhibits viral replication and DNA packaging and precludes the incorporation of portals into capsids [66]. Although these molecules demonstrate activity on HCMV or HSV viral replication, to date, none of them have been used.

Letermovir (AIC246) is a molecule of the quinazoline class having a specific action on HCMV. Letermovir inhibits the cleavage of viral DNA concatemers and the formation of mature HCMV virions by targeting the pUL56 subunit of the viral terminase complex. Preclinical data obtained for this molecule reveal its very strong antiviral activity and low toxicity compared to other antiviral molecules. In phase II trials, letermovir effectively prevented HCMV infection in recipients of allogeneic hematopoietic cell transplants [67]. The phase III trial started in 2014 (by Merck Sharpe and Dohme Corps) was completed at the end of 2016 (ClinicalTrials.gov Identifier: NCT02137772) and published in 2017 [68]. Under the name Prevymis, letermovir was approved (August 2017) by the US Food and Drug Administration. Prevymis is indicated for the prophylaxis of HCMV reactivation and HCMV disease in HCMV-positive adults receiving [R+] allogeneic hematopoietic stem cell transplantation [5,6]. Whereas mutations associated with letermovir resistance map mainly to the pUL56, various letermovir resistance mutations in *UL89* and *UL51* have been identified [69,70,71,72].

Structure and mutagenesis studies revealed essential capsid protein interactions for HHV-8 replication [13]. Polypeptides that mimic the smallest capsid protein to inhibit HHV-8 lytic replication have been developed. In this way, inhibitors mimicking protein interactions may be the molecules of choice for blocking herpesvirus replication. Whether in the form of peptides, polypeptides, antibodies or small drugs, this innovative strategy appears to be promising.

## 6. Concluding Remarks

The herpesvirus capsid assembly and DNA packaging steps are highly specific and have no counterparts in host mammalian cells. They thus represent targets of choice for antiviral drug development. Understanding the HCMV capsid assembly and DNA-packaging process, together with the structure and function of the necessary components, will enable the development of antivirals with high specificity and low toxicity. This knowledge could constitute an essential starting point for the identification of new antiviral targets, and consequently, for the development of molecules for preventive or therapeutic purposes. It would also provide a better understanding of the mechanism of action of molecules in development, such as letermovir.

## Figures and Tables

**Table 1:** Herpesvirus genes involved in capsid assembly and DNA packaging steps.

	α Herpesviruses		β Herpesviruses		γ Herpesviruses		Notes
	HSV-1	VZV	HCMV	HHV-6	HHV-8	EBV	
**Genes encoding capsid proteins**	*UL19*	*ORF40*	*UL86*	*U57*	*ORF25*	*BcLF1*	MCP: component of hexons and pentons
	*UL18*	*ORF41*	*UL85*	*U56*	*ORF26*	*BDLF1*	Triplex dimer linking hexons and pentons together
	*UL38*	*ORF20*	*UL46*	*U29*	*ORF62*	*BORF1*	Triplex monomer linking hexons and pentons together
	*UL35*	*ORF23*	*UL48a*	*U32*	*ORF65*	*BFRF3*	SCP: located on tips of hexons
	*UL26*	*ORF33*	*UL80*	*U53*	*ORF17*	*BVRF2*	Maturational protease: generates mature forms of scaffolding proteins
	*UL26.5*	*ORF33.5*	*UL80.5*	*U53.5*	*ORF17.5*	*BdRF1*	Scaffolding protein removed from capsid during DNA packaging
	*UL6*	*ORF54*	*UL104*	*U76*	*ORF43*	*BBRF1*	Portal protein: forms a dodecametic ring at one of the twelve capsid vertexes, complexed with terminase subunit
**Genes encoding DNA-packaging proteins**	*UL28*	*ORF30*	*UL56*	*U40*	*ORF7*	*BALF3*	Subunit of terminase: “*pac*” site-specific binding, capsid-associated
	*UL15*	*ORF42/45*	*UL89*	*U66*	*ORF29*	*BGRF1/BDRF1*	Subunit of terminase: ATPase subunit, putative nuclease domain
	*UL33*	*ORF25*	*UL51*	*U35*	*ORF67A*	*BFRF1A*	Interacts with terminase and mediates their correct subnuclear localization
	*UL32*	*ORF26*	*UL52*	*U36*	*ORF68*	*BFLF1*	Putative protein involved in capsid localization in the nucleus
	*UL25*	*ORF34*	*UL77*	*U50*	*ORF19*	*BVRF1*	Forms an asymmetric dimer and interacts with pUL17 and the nuclear egress complex
	*UL17*	*ORF43*	*UL93*	*U64*	*ORF32*	*BGLF1*	Anchors terminase to the capsid and is involved in determining the site of the first cleavage reaction

**HSV-1**: human herpesvirus type 1; **VZV**: varicella-zoster virus; **HCMV:** human cytomegalovirus; **HHV-8**: human herpesvirus type 8; **EBV**: Epstein–Barr virus; **MCP**: major capsid protein; **SCP**: smallest capsid protein.

## Data Availability

Not applicable.
